# Dr. Charles H. Richmond

**Published:** 1904-04

**Authors:** 


Dr. Charles H. Richmond, of Livonia, N. Y., died at his home March 2, 1904, aged 63 years. Dr. Richmond graduated in medicine at the University of Buffalo in 1860, and was commissioned assistant surgeon 104th N. Y. Infantry, December 6, 1864. He was mustered out of service with his regiment July 17, 1865. After the war he settled in Livonia and became one of the foremost physicians of his region. He served for 10 years as president of his village and was identified with its growth and improvement.

